# Gender-specific negative association between serum vitamin B12 and testosterone levels in females: the modifying role of BMI in a US adult population

**DOI:** 10.3389/fnut.2025.1579531

**Published:** 2025-07-25

**Authors:** Xin Zhao, Xiaohong Lyu, Hai Wang, Yanan Li, Fei Peng, Yushi Zhang

**Affiliations:** ^1^Department of Urology, Peking Union Medical College Hospital, Chinese Academy of Medical Sciences and Peking Union Medical College, Beijing, China; ^2^Department of Breast Surgery, Peking Union Medical College Hospital, Chinese Academy of Medical Sciences and Peking Union Medical College, Beijing, China

**Keywords:** vitamin B12, testosterone, reproductive health, NHANES, BMI

## Abstract

**Background:**

An increasing number of studies have highlighted the potential role of vitamin B12 in hormonal health, especially its relationship with testosterone levels. Nevertheless, studies examining the association between vitamin B12 and testosterone, particularly in the general population and among women, remain scarce. Using data from National Health and Nutrition Examination Survey (NHANES) 2011–2014, this study aimed to investigate the relationship between vitamin B12 and testosterone levels.

**Method:**

Data for this study were obtained from the NHANES conducted between 2011 and 2014. Multivariable linear regression models were employed to evaluate the associations between vitamin B12 levels and testosterone concentrations in adult participants.

**Results:**

The final study cohort consisted of 4,571 participants with a mean age of 48 ± 18 years. Among them, 50.8% were male, and 49.2% were female. Multivariable weighted linear regression revealed a significant inverse association between serum vitamin B12 levels and testosterone concentrations in females. This association was observed across all three models, including the unadjusted model (*β* = −0.010, 95% CI -0.016 to −0.005), adjusted model I (*β* = −0.007, 95% CI -0.013 to −0.002), and adjusted model II (*β* = −0.008, 95% CI -0.014 to −0.002). Additionally, body mass index (BMI) was identified as an effect modifier, demonstrating a significant negative interaction (*β* = −0.021, 95% CI -0.032 to −0.010) between serum vitamin B12 and testosterone in women aged 20 to 39 years. No statistically significant associations were found between serum vitamin B12 concentrations and total testosterone levels in either the male population or the overall population.

**Conclusion:**

This study demonstrated that serum vitamin B12 levels were negatively associated with testosterone concentrations in the female population, whereas no significant association was observed in males. Moreover, BMI was found to significantly influence the relationship between vitamin B12 and testosterone levels.

## Introduction

Testosterone is a vital sex hormone that plays a significant role in human health and development, influencing both male and female physiology. In recent decades, a concerning decline in testosterone levels among men has been observed, potentially linked to dietary and lifestyle factors (1–3). Among the micronutrients gaining attention for their potential impact on reproductive health is vitamin B12 (cobalamin) (3–5). Vitamin B12 not only plays a critical role in DNA synthesis and cellular metabolism but may also affect hormone levels, including testosterone (6–8). However, current research on the relationship between vitamin B12 and testosterone has primarily focused on infertile men, with limited studies in the general population, particularly among women (5, 9).

Vitamin B12 deficiency affects individuals of all ages, with a higher prevalence in the elderly population. A deficiency in vitamin B12 can result in various neurological and hematological disorders (10). In infertile men, vitamin B12 deficiency has been associated with alterations in testosterone levels, which may further impair fertility (9–11). However, the manifestation of this relationship in the general population, as well as its underlying mechanisms, remains unclear.

Existing research suggests that vitamin B12 may indirectly influence testosterone levels by modulating metabolic pathways and hormone synthesis (12). For instance, vitamin B12 is essential for the methionine cycle, a pathway closely linked to testosterone biosynthesis. Banihani et al. identified a positive correlation between vitamin B12 levels and improved reproductive outcomes, such as enhanced sperm health in men and overall fertility in women. Furthermore, vitamin B12 deficiency can lead to elevated homocysteine levels, a condition associated with cardiovascular diseases and other metabolic disorders (13). In women, although limited research has explored the direct relationship between vitamin B12 and testosterone levels, its impact on overall health and metabolism may indirectly affect hormonal balance (14). Additionally, vitamin B12 deficiency may negatively impact pregnancy and fetal development, underscoring the need for further research into its role in women’s health (15).

Comprehensive research is required to investigate the effects of vitamin B12 on testosterone and overall health in the general population, particularly among reproductive-age men and women. As current studies predominantly focus on infertile men, more inclusive research is necessary to guide nutritional supplementation strategies for diverse populations. This study aimed to investigate the associations between vitamin B12 and testosterone levels in adults, utilizing data from the National Health and Nutrition Examination Survey (NHANES) conducted between 2011 and 2014.

## Method

### Study population

**Figure 1:**
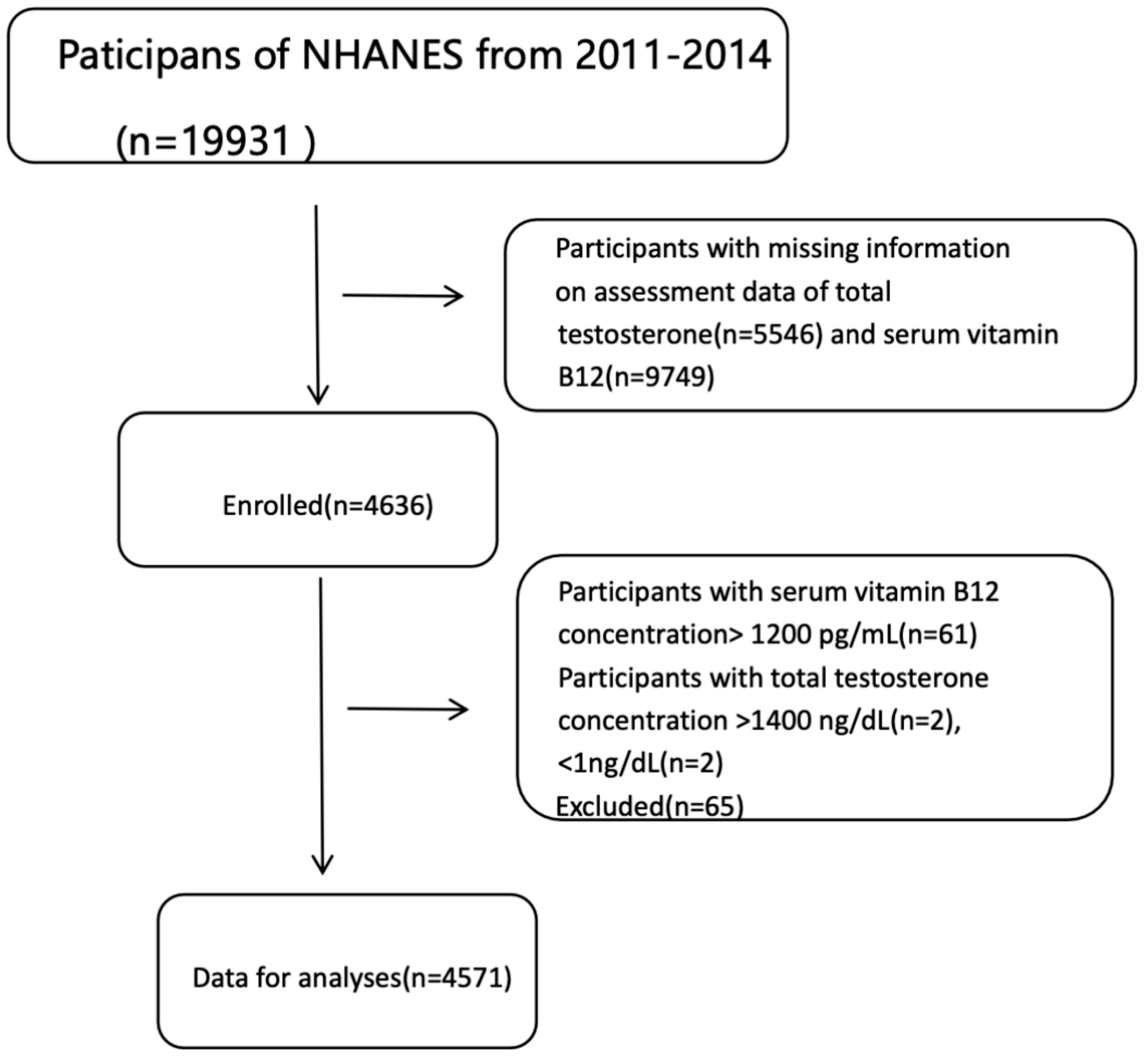
Flow chart of the study population (NHANES 2011–2014).

The study population was derived from the 2011–2014 NHANES dataset, which initially included a total of 19,931 participants. Participants with missing data on total testosterone (*n* = 5,546) or serum vitamin B12 (*n* = 9,749) were excluded from further analysis. Following these exclusions, 4,636 participants remained eligible for inclusion in the study. Further exclusions were applied to participants with serum vitamin B12 concentrations exceeding 1,200 pg./mL (*n* = 61) or total testosterone concentrations exceeding 1,400 ng/dL (*n* = 2) or below 1 ng/dL (*n* = 2). This resulted in an additional exclusion of 65 participants. Ultimately, the final dataset analyzed in this study included 4,571 participants. All NHANES protocols were approved by the National Center for Health Statistics (NCHS) Research Ethics Review Board and the Centers for Disease Control and Prevention (CDC), with written informed consent obtained from all participants for data collection and research purposes (Figure 1).

### Study variables

The primary exposure variable was serum vitamin B12 levels, measured in pmol/L. Serum vitamin B12 levels were measured using the Elecsys Vitamin B12 assay on Elecsys and cobas e immunoassay analyzers at the Division of Laboratory Sciences, National Center for Environmental Health (CDC method Roche E-170 Vitamin B12 ‘ECLIA’ No. 4009.02) (16).

The outcome variable was total testosterone concentration, measured in ng/dL using isotope dilution liquid chromatography–tandem mass spectrometry (ID-LC–MS/MS) on serum specimens at the Division of Laboratory Sciences, National Center for Environmental Health (CDC method No. 1021). Additionally, the following covariates were included in the analysis: age, gender, ethnicity, BMI, ratio of family income to poverty, smoking status, alcohol use, urine albumin, and urine creatinine. Detailed information on these covariates is publicly accessible on the NHANES website.^1^

### Statistical analysis

Following the analytical guidelines provided by NCHS, appropriate sampling weights were applied to ensure national representativeness. Descriptive statistics were used to summarize participant characteristics, including counts and percentages for categorical variables and means (±SD) for continuous variables. Differences among tertiles of serum vitamin B12 levels were analyzed using chi-square tests for categorical variables and analysis of variance (ANOVA) for continuous variables. Serum vitamin B12 concentrations were categorized into tertiles rather than using clinical cutoffs for deficiency or elevated levels. This approach was chosen because clinical cutoffs are based on disease-related epidemiological data and biomarkers unrelated to fertility.

Weighted multivariable regression models were constructed to evaluate the associations between serum vitamin B12 and total testosterone levels. Three models were constructed, including an unadjusted model, a minimally adjusted model (adjusted for age, gender, ethnicity, BMI, and ratio of family income to poverty), and a fully adjusted model. Subgroup analyses stratified by gender, age, and BMI were performed to explore potential effect modifications. Interaction effects between vitamin B12 and total testosterone were tested using interaction terms in the regression models, followed by smooth curve fitting. All analyses were conducted using the statistical software packages R (https://www.r-project.org/, The R Foundation) and Free Statistics Software version 1.3, with *p*-values < 0.05 considered statistically significant.

## Results

Table 1 summarizes the characteristics of the study population (*N* = 4,571), stratified by tertiles of serum vitamin B12 levels. The mean serum vitamin B12 concentrations differed significantly across tertiles: 238.13 pmol/L in the low tertile, 381.73 pmol/L in the middle tertile, and 601.74 pmol/L in the high tertile (*p* < 0.001). The clinical classification of vitamin B12 status showed that 2.01% of participants were deficient, 91.53% were within the optimal range, and 6.54% had elevated levels. The gender distribution was nearly balanced, with 50.80% male and 49.20% female participants. The mean age of participants was 48 years, with significant differences observed across tertiles (*p* = 0.006). The mean body mass index (BMI) was 28.87 kg/m^2^, with the highest tertile exhibiting the lowest mean BMI (28.30 kg/m^2^) compared to the lowest tertile (29.46 kg/m^2^) (*p* < 0.0001). The ethnic distribution revealed that non-Hispanic whites constituted the largest group (38.07%), with significant differences observed across tertiles (*p* = 0.0001). For lifestyle factors, the majority of participants were nonsmokers (56.5%) and reported consuming alcohol (66.4%).

**Table 1:** Subject characteristics in this study.

Characteristics	Subclasses	*N* (%)	Mean ± SD	Tertiles of serum vitamin B12			*p*-value
				Low	Middle	High	
		4,571		1,515	1,529	1,527	
Serum Vitamin B12 (pmol/L), mean ± SD			407.6 ± 166.7	238.1 ± 52.0	381.73 ± 41.3	601.7 ± 109.1	<0.001
Vitamin B12 clinical status, *N* (%)	Deficient	92(2.0)					
	Optimal	4,184(91.5)					
	Elevated	295(6.5)					
Gender, *N* (%)	Male	2,322(50.8)		774(51.1)	832(54.4)	716(46.9)	0.0002
	Female	2,249(49.2)		741(48.9)	697(45.6)	811(53.1)	
Age, mean ± SD			48.06 ± 17.6	48.3 ± 17.2	47.0 ± 17.6	49.0 ± 18.0	0.006
Age group class, *N* (%)	20–39	1,515(33.1)		536 (31.9)	540 (35.6)	439 (32.0)	0.002
	40–59	1,529(33.5)		600 (35.7)	502 (33.1)	427 (31.1)	
	60+	1,527(33.4)		544 (32.4)	476 (31.4)	507 (36.9)	
Body Mass Index(BMI), kg/m^2^, mean ± SD			28.9 ± 6.8	29.5 ± 7.1	28.9 ± 6.7	28.3 ± 6.3	<0.001
BMI Group class, *N* (%)	≤24.9	1,399(30.6)		412(29.5)	481(34.4)	506(36.1)	
	25–29.9	1,478(32.3)		512(34.6)	485(32.8)	481(32.6)	
	≥30	1,632(35.7)		570(34.9)	545(33.4)	517(31.7)	
Ethnicity, *N* (%)	Non-Hispanic White	1740(38.1)		636(42.0)	585(38.2)	519(34.0)	0.0001
	Non-Hispanic Black	1,134(24.8)		335(22.1)	374(24.5)	425(27.8)	
	Mexican-American	465(10.2)		139(9.2)	173(11.3)	153(10.0)	
	Others	1,232(27.0)		405(26.7)	397(26.0)	430(28.2)	
Ratio of family income to poverty, mean±SD			2.213 ± 1.63	2.197 ± 1.61	2.209 ± 1.64	2.233 ± 1.64	0.85
Smoking status, *N* (%)	Smoker	1989(43.5)		687(45.4)	690(45.13)	612(40.1)	0.004
	Non-Smoker	2,582(56.5)		828(54.7)	839(54.9)	915(59.9)	
Alcohol use, *N* (%)	Drinker	3,034(66.4)		1,016(67.1)	1,062(69.5)	956(62.6)	0.001
	Non-Drinker	1,048(22.9)		337(22.2)	310(20.3)	401(26.3)	
	Unspecified	489(10.7)		162(10.7)	157(10.3)	170(11.1)	
Total testosterone ng/dL, mean ± SD			221.4 ± 234.3	220.2 ± 229.6	237.2 ± 237.6	206.6 ± 234.5	0.001
Urine albumin ug/mL, mean ± SD			42.70 ± 245.1	33.2 ± 140.8	48.8 ± 328.2	46.0 ± 227.9	0.074
Urine creatinine mg/dL, mean ± SD			126.0 ± 82.7	127.3 ± 82.0	130.7 ± 82.8	119.80 ± 83.2	0.001

Summarizes the demographic and laboratory characteristics of the study population (*N* = 4,571) based on the NHANES 2011–2014 database, specifically focusing on serum Vitamin B12 levels and associated factors. Tertile of serum vitamin B12 concentration cutoffs are <313.7 pmol/L for the lowest tertile, ≥313.7 to <459.8 pmol/L for the mid-tertile, and ≥459.8 pmol/L for the highest tertile, respectively. Serum vitamin B12 concentration cutoffs are <148 pmol/L for deficient, ≥148 to <701 pmol/L for optimal, and ≥701 pmol/L for elevated, respectively. Comparisons of laboratory and demographic data between quartile levels were performed using one way ANOVA for continuous variables the chi-square test. All *p*-values was calculated by weighted. Statistical significance was set at *p* < 0.05.

Analyzing the data using analysis of variance and chi-square tests, we observed significant variations across serum vitamin B12 tertile groups for total testosterone. These results highlight the diverse characteristics of the population in relation to serum vitamin B12 levels.

Table 2 presents the relationship between serum vitamin B12 levels and testosterone concentrations, evaluated using multivariable regression models with stratification by gender, age, and BMI. No statistically significant association was observed between serum vitamin B12 concentrations and total testosterone levels in either univariate or multivariate regression analyses. The unadjusted model showed a *β* value of −0.029 (95% CI -0.068 to 0.010, *p* = 0.147), indicating no significant association between serum vitamin B12 and testosterone levels. After adjustment in Model I, the β coefficient was −0.006 (95% CI -0.027 to 0.016, *p* = 0.607). Similarly, in Model II, the β coefficient remained −0.006 (95% CI -0.028 to 0.017, *p* = 0.631), both indicating no statistical significance. Furthermore, no significant association was observed between serum vitamin B12 tertiles and testosterone levels in any of the models.

**Table 2:** Association between for association between serum vitamin B12 concentration (pmol/L) and total testosterone(ng/dL) concentration.

	Non-adjusted *β* (95%Cl)	*P*-value	Adjusted Model I *β* (95%Cl)	Adjusted *p*-value	Adjusted Model II *β* (95%Cl)	Adjusted *p*-value
Serum vitamin B12	−0.029 (−0.068, 0.010)	0.147	−0.006 (−0.027, 0.016)	0.607	−0.006 (−0.028, 0.017)	0.631
Low	Reference		Reference		Reference	
Mid	14.840 (−1.381, 31.060)	0.073	0.812 (−7.999, 9.622)	0.85	−0.604 (−9.729, 8.522)	0.890
High	−6.792 (−23.002, 9.417)	0.411	0.173 (−8.688, 9.035)	0.96	−2.434 (−11.630,6.763)	0.600
*p*-value for trend	0.417		0.968		0.435	
Stratified by age	−0.027 (−0.066, 0.012)	0.169	−0.007 (−0.028, 0.015)	0.538	−0.006 (−0.028, 0.016)	0.594
20–39	0.084 (0.012, 0.156)	0.022	−0.013(−0.052, 0.025)	0.499	−0.020(−0.058, 0.019)	0.315
40–59	−0.060 (−0.126, 0.006)	0.075	−0.007(−0.041, 0.027)	0.671	−0.010(−0.046, 0.026)	0.595
60+	−0.101 (−0.165, −0.037)	0.0019	−0.022(−0.060, 0.016)	0.262	−0.015(−0.057, 0.027)	0.479
Stratified by gender	0.006 (−0.016, 0.028)	0.589	−0.006(−0.027, 0.016)	0.601	−0.005(−0.028, 0.017)	0.631
Male	0.024 (−0.020, 0.068)	0.287	−0.008(−0.048, 0.032)	0.691	−0.006(−0.048, 0.036)	0.763
Female	−0.010 (−0.016, −0.005)	<0.0001	−0.007(−0.013,-0.002)	0.013	−0.008(−0.014, −0.002)	0.009
Stratified by BMI	−0.042(−0.081,-0.004)	0.032	−0.001(−0.023,0.020)	0.899	−0.001(−0.023,0.021)	0.917
≤24.9	−0.065 (−0.144, 0.014)	0.106	−0.003(−0.042, 0.036)	0.879	0.013(−0.029, 0.054)	0.543
25–29.9	−0.030 (−0.100, 0.039)	0.391	0.004 (−0.035, 0.044)	0.841	−0.006(−0.047, 0.035)	0.768
≥30	−0.033 (−0.087, 0.020)	0.221	0.007 (−0.022, 0.037)	0.641	0.001(−0.030, 0.032)	0.943

Unadjusted model adjust for none. Adjusted Model I adjusted for covariates: age, gender, ethnicity, BMI, ratio of family income to poverty. Model II adjusted for covariates: age, gender, ethnicity, BMI, ratio of family income to poverty, smoking status, alcohol use, albumin urine, urine creatinine. In the subgroups analysis were stratified by age, gender and BMI, the model was not adjusted for the stratification variable itself.

After adjusting for potential confounding factors, no significant association was observed between serum vitamin B12 levels and testosterone concentrations across all age groups. In the unadjusted model, the *β* coefficient for the 20–39 years age group was 0.084 (95% CI 0.012 to 0.156, *p* = 0.022), indicating a significant positive correlation. For the 40–59 years age group, the *β* coefficient was −0.060 (95% CI -0.126 to 0.006, *p* = 0.075), which was close to statistical significance. For individuals aged 60 years and older, the *β* coefficient was −0.101 (95% CI -0.165 to −0.037, *p* = 0.0019), indicating a significant negative correlation. However, after adjustment for potential confounding factors, the observed associations were no longer statistically significant.

In the gender-stratified analysis, a negative association between serum vitamin B12 levels and testosterone concentrations was observed in all three models for females, but not for males. After adjusting for potential confounding factors, the *β* coefficient for the male group was −0.006 (95% CI –0.048 to 0.036, *p* = 0.763), indicating no statistical significance. In contrast, the β coefficient for the female group was −0.008 (95% CI –0.014 to −0.002, *p* < 0.009), indicating a statistically significant negative correlation. The overall analysis yielded a *β* coefficient of −0.005 (95% CI –0.028 to 0.017, *p* = 0.631), which was not statistically significant.

In the BMI-stratified analysis, a significant negative association was observed in the unadjusted model for the overall population. The *β* coefficient was −0.042 (95% CI –0.081 to 0.004); however, this association was not observed in the adjusted models across all BMI groups. Furthermore, this negative association was not evident across any of the three BMI categories.

Table 3 summarizes the interaction *p*-values and the results of adjusted linear regression models examining the relationship between serum vitamin B12 concentrations (pmol/L) and total testosterone levels (ng/dL) across different age groups and BMI categories for both males and females. The potential effect modification by age and BMI was examined. In males, the relationship between serum vitamin B12 concentrations and testosterone levels was not statistically significant across different age and BMI groups, regardless of whether in the unadjusted or adjusted models. For the age group 20–39 years, the *β* coefficient was 0.053 (*p* = 0.1654); for the 40–59 years group, it was 0.019 (*p* = 0.6517); and for individuals aged 60 years and older, it was −0.012 (*p* = 0.7479), all indicating no statistically significant associations. The interaction *p*-value across age groups was 0.473. Similarly, in the BMI-stratified analysis, the β coefficients for BMI ≤ 24.9, 25–29.9, and ≥30 were 0.006 (*p* = 0.8648), 0.005 (*p* = 0.8949), and 0.004 (*p* = 0.9115), respectively. The interaction p-value was 0.999, further confirming that BMI does not significantly modify the relationship between serum vitamin B12 concentrations and testosterone levels.

**Table 3:** The interaction *p*-value assesses the adjusted linear regression model examining the relationship between serum vitamin B12 concentration (pmol/L) and total testosterone(ng/dL) concentration in male and female.

Gender	Age			*P* interaction for age
	20–39	40–59	60+	
Male				
Non-adjusted β (95%Cl)	0.053 (−0.022, 0.127)	0.019 (−0.062, 0.099)	−0.012 (−0.084, 0.060)	0.473
*p*-value	0.1654	0.6517	0.7479	
Adjusted Model β (95%Cl)	−0.015 (−0.086, 0.056)	0.035 (−0.042, 0.112)	−0.018 (−0.090, 0.053)	0.545
*p*-value	0.6828	0.3748	0.6132	
Female				
Non-adjusted β (95%Cl)	−0.011 (−0.021, −0.001)	−0.009 (−0.019, 0.000)	−0.005 (−0.014, 0.005)	0.627
*p*-value	0.0279	0.0506	0.3476	
Adjusted Model *β* (95%Cl)	−0.011 (−0.021, −0.000)	−0.009 (−0.019, 0.002)	−0.005 (−0.016, 0.006)	0.734
*p*-value	0.0465	0.1004	0.3768	

**Gender**	**BMI**			*P* interaction for age
	≤24.9	25–29.9	≥30	
Male				
Non-adjusted β (95%Cl)	0.006 (−0.065, 0.078)	0.005 (−0.064, 0.073)	0.004 (−0.074, 0.083)	0.999
*p*-value	0.8648	0.8949	0.9115	
Adjusted Model β (95%Cl)	0.035 (−0.039, 0.108)	−0.018 (−0.088, 0.053)	−0.025 (−0.105, 0.055)	0.478
*p*-value	0.3528	0.6265	0.5413	
Female				
Non-adjusted β (95%Cl)	−0.022 (−0.032, −0.011)	−0.014 (−0.025, −0.004)	0.001 (−0.008, 0.010)	0.003
*p*-value	<0.0001	0.0074	0.7938	
Adjusted Model β (95%Cl)	−0.021 (−0.032, −0.010)	−0.008 (−0.020, 0.003)	0.002 (−0.008, 0.011)	0.010
*p*-value	0.0002	0.1593	0.7452	

Interaction P for the adjusted linear regression model assessing the association between serum vitamin B12 and total testosterone with effect modification by age and BMI in male and female. Adjusted Model adjusted for covariates included age, gender, ethnicity, BMI, ratio of family income to poverty, smoking status, alcohol use, urine albumin, and urine creatinine.

In females, a significant negative correlation was observed between testosterone and vitamin B12 in the age group 20–39 years, with a *β* coefficient of −0.011 (*p* = 0.0465). In the age group 40–59 years, this negative correlation approached significance (*β* coefficient = −0.009, *p* = 0.1004) but did not reach statistical significance. However, the interaction *p*-value across age groups was 0.627, indicating no statistically significant difference; thus, age cannot be considered an important moderating factor.

**Figure 2:**
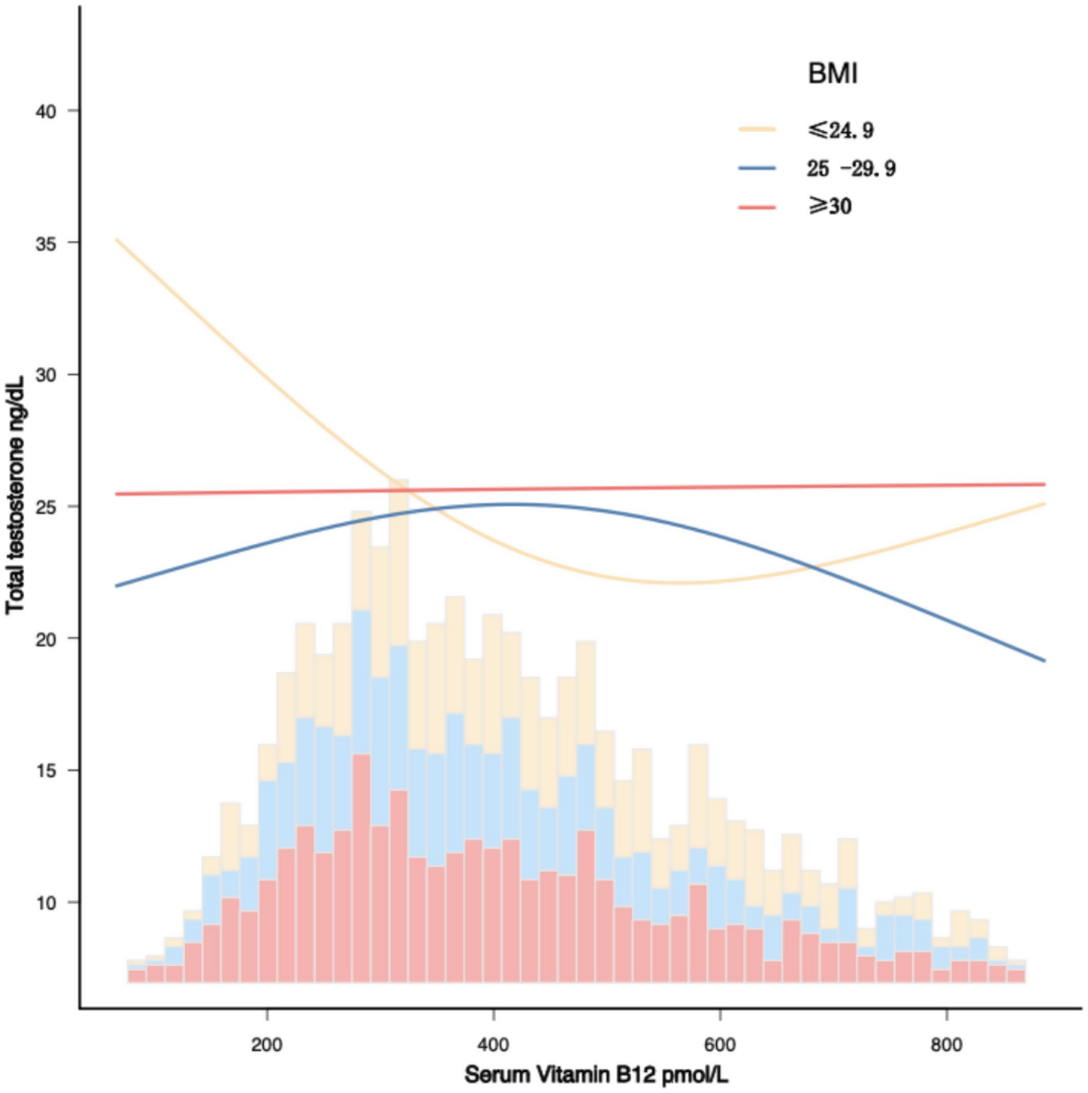
The association between serum vitamin B12 and total testosterone by BMI in female. The adjustment covariates included age, gender, ethnicity, ratio of family income to poverty, smoking status, alcohol use, urine albumin, urine creatinine.

Most importantly, a significant negative correlation between vitamin B12 and testosterone was observed, particularly in the BMI ≤ 24.9 group, where the *β* coefficient was −0.022 (*p* < 0.0001). In the BMI-stratified analysis, the *β* coefficients for BMI ≤ 24.9, 25–29.9, and ≥30 were −0.021 (*p* < 0.0001), −0.008 (*p* = 0.1593), and 0.002 (*p* = 0.7452), respectively, with an interaction *p*-value of 0.010. The BMI effect is also illustrated in Figure 2. Thus, BMI acts as an effect modifier, with a significant negative interaction observed between serum vitamin B12 and testosterone in females.

## Discussion

This study explored the relationship between serum vitamin B12 levels and testosterone concentrations in a large, diverse cohort of U.S. adults. Our findings revealed a significant negative association between serum vitamin B12 levels and testosterone concentrations in females, particularly among those with a BMI ≤ 24.9. In contrast, no such association was observed in males. Furthermore, BMI was identified as a significant effect modifier in females, with stronger negative associations observed in normal-weight individuals. These results suggest that vitamin B12 may play a role in hormonal regulation, particularly in women.

Previous studies examining the relationship between vitamin B12 and testosterone have primarily focused on male populations (16), particularly infertile men (9). For instance, Isoyama et al. reported that vitamin B12 supplementation improved sperm parameters (17). Similarly, Rastegar et al. found a positive association between vitamin B12 and testosterone levels in infertile men (9). However, our study, which included a general population sample, did not observe a significant association in males. In contrast, our findings revealed a negative association in females, a relationship that has been largely unexplored in the existing literature. This underscores the importance of considering gender-specific differences when investigating the effects of vitamin B12 on hormonal health.

The observed negative association between serum vitamin B12 and testosterone levels in females may be explained by several potential mechanisms. Vitamin B12 is essential for DNA synthesis (8), cellular metabolism (18), and methylation processes (19), which are critical for hormone biosynthesis (20). It is possible that vitamin B12 indirectly influences testosterone levels by modulating estrogen metabolism (21–23). Estrogen, which is typically higher in females, may suppress testosterone synthesis (24), and vitamin B12 might amplify this effect by enhancing estrogen activity or synthesis (25). Additionally, vitamin B12’s role in reducing homocysteine levels could influence metabolic pathways linked to hormone regulation (26). Vitamin B12 might amplify this effect by enhancing estrogen activity or synthesis through methylation-dependent pathways (27). Additionally, sex hormone-binding globulin (SHBG), a glycoprotein that binds to sex hormones and regulates their bioavailability, may play a role in mediating the observed relationship (28). Elevated vitamin B12 levels could influence SHBG production, thereby altering the balance between free and bound testosterone. This hypothesis aligns with previous research suggesting that SHBG levels are sensitive to metabolic and nutritional factors, including vitamin status (29). Furthermore, the role of vitamin B12 in reducing homocysteine levels may also be relevant (30), as elevated homocysteine has been implicated in disruptions to hormone synthesis and metabolic homeostasis (31).

The findings in this study highlight the potential importance of vitamin B12 in maintaining hormonal balance in females, particularly in those with normal BMI. Elevated testosterone levels in women are associated with various health conditions, including polycystic ovary syndrome (PCOS) and insulin resistance (32, 33). Understanding the role of vitamin B12 in hormonal regulation could inform nutritional interventions aimed at improving reproductive and metabolic health. Additionally, the gender-specific and BMI-dependent effects observed in this study underscore the need for personalized approaches to nutritional supplementation and hormonal health management.

This study has several limitations that should be acknowledged. While it utilized a large and nationally representative sample from NHANES, ensuring the generalizability of the findings to the U.S. adult population, the cross-sectional nature of the data restricts our ability to infer causality or the directionality of the observed associations. Although rigorous statistical methods, including multivariable regression and stratified analyses, were employed to enhance the reliability and robustness of the results, the study design does not allow us to determine whether altered vitamin B12 levels directly influence testosterone concentrations or whether hormonal changes affect vitamin B12 metabolism. Furthermore, the observed effects could be mediated by other unmeasured biological or lifestyle factors. Despite these limitations, the study provides novel insights into the gender-specific relationship between vitamin B12 and testosterone levels, with BMI identified as a significant effect modifier in females. These findings contribute to the limited existing literature and provide a foundation for future research on the role of vitamin B12 in hormonal health. Longitudinal studies are needed to establish causal relationships, explore the temporal dynamics between vitamin B12 and testosterone levels, and confirm and expand upon these results.

Future studies should aim to elucidate the mechanistic links between vitamin B12 and testosterone levels, particularly the role of estrogen metabolism and other hormonal pathways. Longitudinal studies are needed to establish causal relationships and explore the effects of vitamin B12 supplementation on hormonal health. Additionally, research should investigate how metabolic factors, such as insulin resistance and lipid profiles, interact with vitamin B12 to influence hormonal regulation. Expanding the study population to include diverse ethnic and geographic groups would also enhance the generalizability of the findings.

## Conclusion

Our findings highlight the critical role of vitamin B12 in maintaining hormonal balance, particularly in females. The interplay between vitamin B12, BMI, and testosterone levels suggests that nutritional and metabolic factors may significantly influence hormonal health, underscoring the importance of personalized approaches in managing reproductive health.

## Data Availability

Publicly available datasets were analyzed in this study. This data can be found at: https://www.cdc.gov/nchs/nhanes/index.html.

## Footnotes

^1^ www.cdc.gov/nchs/nhanes/
